# Prevalences and trends of chronic diseases in Shaanxi Province, China: Evidence from representative cross-sectional surveys in 2003, 2008 and 2013

**DOI:** 10.1371/journal.pone.0202886

**Published:** 2018-08-23

**Authors:** Sha Lai, Jianmin Gao, Zhongliang Zhou, Xiaowei Yang, Yongjian Xu, Zhiying Zhou, Gang Chen

**Affiliations:** 1 School of Public Policy and Administration, Xi’an Jiaotong University, Xi’an, Shaanxi, China; 2 School of Public Health, Health Science Center, Xi’an Jiaotong University, Xi’an, Shaanxi, China; 3 Centre for Health Economics, Monash University, Clayton, VIC, Australia; Shanghai Diabetes Institute, CHINA

## Abstract

**Objectives:**

Non-communicable diseases, which can refer to chronic diseases that are not caused by infectious agents and can endure for a long time, are currently regarded as a critical public health problem in China. This study aimed to estimate the prevalences of self-reported physician-diagnosed chronic diseases among urban and rural populations aged 15 years and older in Shaanxi Province, China, during 2003−2013 and explore how these changes differ by subpopulation.

**Methods:**

Three independent cross-sectional surveys were implemented in 2003, 2008 and 2013 in Shaanxi Province. A multistage stratified cluster random sampling method was used in each wave to collect representative samples. In total, 10,568 residents in 2003, 15,453 in 2008 and 48,808 in 2013 were included in this analysis. Information on self-reported physician-diagnosed chronic diseases was collected using face-to-face interviews in each survey. Multilevel Poisson regression with robust error variance was employed to calculate the adjusted prevalence ratios to estimate the relative change in chronic conditions in 2008 and 2013, compared to that in 2003.

**Results:**

In 2013, 23.9%/22.1% of urban/rural residents, respectively, reported having at least one chronic condition, which represents an increase from 17.0%/15.1%, respectively, in 2008 and 12.8%/10.9%, respectively, in 2003. Adjusted for socio-demographic characteristics, the prevalence of chronic diseases was significantly higher in 2013 than that in 2003. Among the chronic diseases studied, the prevalence of hypertension and diabetes has increased dramatically over a decade. The increase in chronic diseases occurred mainly among the middle-aged and elderly.

**Conclusions:**

Chronic diseases are highly prevalent and continuously increasing in the adult population in Shaanxi Province from 2003 to 2013. Given its large aging population, China may face a greater chronic disease burden. A national chronic disease surveillance system and screening program should be established to acquire comprehensive information regarding the distribution and trends of chronic diseases.

## Introduction

In China, non-communicable diseases (NCDs) are emerging as a significant cause of poor health, with 70% of disease burden and 80% of deaths being attributed to NCDs [1]. Ageing, unhealthy lifestyles or diets and environmental deterioration all contribute to the prevalence of NCDs, along with the rapid economic growth and the acceleration of industrialization and urbanization in China [2–4]. NCDs are currently regarded as a critical public health problem in China. In 2002, The National Center for Chronic and Non-communicable Disease Control and Prevention was established, with the core missions of providing scientific evidence/consultation for developing policies and regulations and defining national plans, strategies and technical standards for major NCDs [5]. The provincial-level disease control agencies, primary-level healthcare institutions and general hospitals in province, city and district units constitute the local prevention and control network. A multilayered pattern of NCD control and prevention has been developed. A national NCD control network and service system was set up gradually, performing classification management to main chronic diseases using a range of comprehensive measures. Several policies and strategies of NCD control and prevention in China have been implemented at both the national and regional levels.

For NCD patients, a disease-oriented approach has been adopted, such as implementing health management plans for patients with hypertension or diabetes (including measuring blood pressure and glucose periodically and distributing complimentary hypotensors or hypoglycaemic medications by community doctors). Primary health care has been strengthened for the general population. In 2007, the “China Healthy Lifestyle for All” project was launched to promote healthy lifestyles and prevent diseases at the individual and population levels [6]. Special projects have also been established for high-risk groups. For instance, the Rural Cervical and Breast Cancer Screening Project was initiated in 2009 to provide free check-ups for breast and cervical cancer among women aged 35−59 years in rural areas. Despite these efforts, the increased prevalence of chronic diseases at the national level could not be prevented [7]. The mortality rate attributed to chronic diseases in China is higher than that in some developed countries [8].

Most of the previous large-scale survey studies performed among Chinese populations with NCDs focused on one or more specific diseases based on on-site diagnosis and questionnaire surveys, such as hypertension and diabetes [9–13]. Other studies described the changing patterns of chronic diseases based on surveillance data [14, 15]. Few studies have been published to investigate the temporal changes of multiple chronic diseases based on large-population, individual-level data and multiple representative cross-sectional surveys, particularly in the last 10 years, during which developing countries are undergoing health transitions. In China, as the chronic disease information system is far from perfect, it is still a challenge to accurately measure the prevalences of many NCDs. Additionally, given the uniqueness of social structure, economic profiles and lifestyles in local areas, there may be disparities in epidemiological features of chronic diseases between different provinces and regions. This is specifically the case for Shaanxi Province, China, where health resources are relatively deficient, and both economic and social developments lag behind the Central and Eastern regions.

In epidemiology, prevalence, which is a key indicator of diseases, quantifies how widespread the disease is in the population. In this study, we aimed to present the most updated prevalences of chronic diseases in urban and rural populations aged 15 years and older in Shaanxi Province, using three representative and comparable cross-sectional household health surveys conducted in 2003, 2008 and 2013. Furthermore, this study investigated the associations between socio-demographic characteristics and chronic disease prevalences.

## Materials and methods

### Study population

The data in the study were drawn from the third, fourth and fifth National Health Services Survey (NHSS) in Shaanxi Province conducted in 2003, 2008 and 2013, respectively. The NHSS, organised by the National Health Commission of the People’s Republic of China (NHPC), is the largest health survey in China and, since 1993, is conducted every 5 years. The NHSS questionnaire mainly includes demographic information, economic status, health status and lifestyle characteristics. In this study, face-to-face interviews were conducted for each household.

A multistage stratified cluster random sampling method was used in each wave to collect representative samples in urban and rural regions, respectively. In each wave, a new set of participants was sampled from Shaanxi Province. In brief, 41/44/32 counties in rural areas or districts in urban areas were stratified in 2003/2008/2013 in Shaanxi Province, among which 60/75/160 townships (sub-districts) were randomly selected in sampled counties or districts. Next, 105/150/320 villages (communities) were randomly selected from townships in 2003/2008/2013. Finally, 3,967/5,960/20,700 households were interviewed in 2003/2008/2013. In total, 13,506 residents in 2003 (6,344 urban and 7,162 rural), 18,290 in 2008 (7,948 urban and 10,342 rural) and 57,529 in 2013 (21,325 urban and 36,204 rural) were identified.

Shaanxi Province had a population of 36.7 million in 2003 (64.5% lived in rural areas), 37.2 million in 2008 (57.9% lived in rural areas) and 37.6 million in 2013 (48.7% lived in rural areas), among which men accounted for 51.3%, 51.4% and 51.6%, respectively [16]. In terms of the population's age structure, the proportion of the population aged 65 years and older in total population in Shaanxi Province grown to 9.4% in 2013, from 9.0% in 2008 and 7.8% in 2003; the proportion of the population aged 15−64 years has also grown to 76.3% in 2013, from 73.3% in 2008 and 71.4% in 2003 [16]. The Myer’s Blended Index [17] was 1.95 in 2003 (2.18 in urban and 1.99 in rural), 1.67 in 2008 (1.79 in urban and 2.16 in rural) and 1.63 in 2013 (1.85 in urban and 1.76 in rural), indicating that the respondents were representative of Shaanxi Province for age in all three waves.

Considerable quality control measures were implemented during data collection [18]. First, the interviewers were medical workers from local villages or communities and were trained by their supervisors (who were doctors from higher-level health institutions) before conducting the interviews. Second, notification was given prior to the survey to ensure that the respondents knew when the interview would take place. If the respondents were not at home, the interviewers would return for a maximum of three times to complete the survey. Third, the respondents were required to answer the questions by themselves, except for children under the age of 6 years. Fourth, if there were missing information and logical errors in the questionnaire, a resurvey was required the next day. Moreover, 5% of the households were revisited, and eight questions were re-interviewed to check for consistency. Based on these efforts, high response rates (>85%) and high consistency between survey and re-interviewed survey (>95%) were achieved in all three wave surveys. The self-response rates were also above 75% in all three wave surveys.

As residents under the age of 15 years did not answer questions about chronic disease and some socio-economic characteristics (e.g., marital status, education and career status), in the surveys, we only focused on residents aged 15 years and above. After excluding those respondents who had missing values in key variables (<0.4%), a total of 10,568 residents in 2003 (49.5% urban), 15,453 in 2008 (44.9% urban) and 48,808 in 2013 (36.8% urban) were adopted for analysis in this study.

### Measures

The presence of physician-diagnosed chronic diseases in the last 6 months was the primary outcome variable in this study. The questions were as follows: “Have you ever been diagnosed with any chronic diseases during the last 6 months?” If the answer is “yes,” continue with questions, for example: “According to severity, please list the names of diseases; if there were more than one disease, please name up to three chronic diseases.” In the NHSS, the chronic diseases refer to either: 1) chronic diseases diagnosed in the last 6 months or 2) chronic diseases diagnosed 6 months ago and earlier, but the patient is receiving ongoing treatment or received treatment during the period since the onset of the disease and until the last 6 months. The reported chronic diseases were coded by NHPC according to the International Classification of Diseases, 10th revision coding system [19], which is the international standard diagnostic classification for clinical use, general epidemiological purposes and health management purposes.

Socio-demographic characteristics included rural/urban residency, gender, age (15−34, 35−54, 55−64 and 65 years and older), economic status, education level (primary school or below, junior middle school and high school or above) and occupational status (employed and unemployed group). Economic status was defined by self-reported household consumption expenditure, which was adjusted according to the household scale, and further divided into five percentiles, with the first quintile representing the poorest income group and the fifth quintile representing the richest. It is proposed that self-reported expenditures are better indicators for household economic status in developing countries [20]. Lifestyle characteristics includes smoking (never-smokers, current smokers and ex-smokers) and drinking frequency (almost never drinking, 1–2 times per week and 3 times and above per week).

### Statistical analysis

The statistical analyses were conducted separately for urban and rural populations, considering the urban-rural duality structure in China.

For the first stage of the analysis, the prevalences of NCDs were plotted for three survey periods (2003, 2008 and 2013) and were analysed according to the socio-demographic characteristics. Crude prevalences of NCDs by socio-demographic characteristics were produced separately for each survey year. Age-adjusted prevalences of NCDs for six of the most common NCDs in 2003, 2008 and 2013 were presented, with adjustment according to the data of the 2010 national population census in Shaanxi Province.

In the second stage of the analysis, multilevel Poisson regressions with robust error variance were used to examine the factors associated with chronic diseases. Given that binary outcomes (presence of chronic diseases in this case) have high prevalence, we estimated the adjusted prevalence ratios (PRs) instead of the odd ratios [21, 22]. The PRs can be interpreted as the prevalence rate ratio between the reference group and another group after adjusting for confounding variables. A PR value >1 indicated higher risk factor prevalence than the reference group, and vice versa. Robust Poisson regression, for which the link function is logarithm, has been suggested as a good alternative for estimating PR adjusted for confounding variables in a cross-sectional study, and does not have any convergence difficulty [23]. To account for the intra-cluster correlation owing to the multistage stratified cluster random sampling method, a multilevel analysis was adopted, with the townships/sub-districts level specified as level three, villages/communities as level two and individuals nested within these areas as level one unit.

In the final stage of the analysis, we estimated the relative change over 10 years with prevalences of NCDs in gender and age subpopulations by creating subpopulation datasets for all years and performing multilevel Poisson regressions with robust error variance adjusting for gender, age and other socio-demographic characteristics. In this stage, we explored how these changes differed by gender and age.

Sampling weights were incorporated in calculating prevalences and standard errors and estimating multilevel regression models to reflect the survey methodology. All of the statistical analyses were conducted using Stata software version 14.0. P<0.05 was considered to be statistically significant.

### Ethical statements

To protect the privacy of the respondents, the organiser (NHPC) was committed to preserving anonymousity in the analysis in accordance with the Statistics Law of People’s Republic of China. Each respondent agreed to participate in this interview and provided informed consent. The Ethics Committee of Xi’an Jiaotong University Health Science Center approved this study (approval number 2015–644).

## Results

Table 1 shows the respondents’ socio-demographic and lifestyle characteristics in each wave by region. Except for gender, statistically significant differences existed in all characteristics and behaviours across three waves in either urban or rural region (P<0.05). Comparing the smoking behaviours of respondents in urban and rural regions, the percentage of the respondents who were current smokers steadily increased in the urban region but remained stable in the rural region between 2003 and 2013 (from 23.5% to 27.1% and from 29.3% to 30.1%, respectively). However, the proportions of ex-smokers were also increased in urban and rural regions (from 1.8% to 3.8% and from 1.1% to 2.6%, respectively). With regard to drinking frequency, the percentages of low-frequency drinkers (i.e., drinking 1−2 times per week) were decreased in urban and rural regions (from 12.6% to 5.0% and from 12.5% to 4.6%, respectively) over the decade.

**Table 1:** Characteristics of respondents aged 15 years and older in 2003, 2008 and 2013.

	Urban				Rural			
	2003, N (%)^a^	2008, N (%)^a^	2013, N (%)^a^	p^b^	2003, N (%)^a^	2008, N (%)^a^	2013, N (%)^a^	p^b^
Total	5,236	6,934	17,973		5,332	8,519	30,835	
Gender								
Men	2,570 (49.2)	3,361 (48.5)	8,653 (48.1)	0.419	2,717 (50.7)	4,264 (50.0)	15,235 (49.6)	0.347
Women	2,666 (50.8)	3,573 (51.5)	9,320 (51.9)		2,615 (49.3)	4,255 (50.0)	15,600 (50.4)	
Age								
15–34	1,762 (33.6)	2,007 (29.1)	4,734 (24.5)	<0.001	2,029 (38.0)	2,558 (30.5)	6,811 (22.0)	<0.001
35–54	2,039 (39.0)	3,003 (43.4)	7,101 (39.6)		2,307 (43.4)	3,656 (42.4)	13,333 (43.3)	
55–64	629 (12.1)	974 (14.0)	2,990 (16.9)		559 (10.5)	1,276 (14.9)	5,920 (19.2)	
65 years or older	806 (15.3)	950 (13.6)	3,148 (19.0)		437 (8.2)	1,029 (12.2)	4,771 (15.5)	
Economic status								
Lowest 20%	1,057 (20.0)	1,387 (20.1)	3,601 (19.6)	-	1,071 (19.3)	1,781 (21.2)	6,193 (20.3)	-
Lower 20%	1,050 (20.2)	1,391 (19.8)	3,591 (20.4)		1,059 (20.0)	1,718 (19.5)	6,150 (20.0)	
Middle 20%	1,032 (19.8)	1,384 (19.9)	3,597 (20.4)		1,076 (21.1)	1,656 (19.1)	6,159 (19.8)	
Higher 20%	1,045 (19.9)	1,386 (19.9)	3,592 (20.4)		1,056 (20.5)	1,686 (19.4)	6,168 (19.8)	
Highest 20%	1,052 (19.9)	1,386 (20.3)	3,592 (19.2)		1,070 (19.1)	1,678 (20.6)	6,165 (20.1)	
Education level								
Primary school or below	1,142 (22.0)	1,120 (16.2)	4,741 (22.4)	<0.001	2,449 (44.4)	4,049 (46.9)	14,554 (46.5)	<0.001
Junior high school	1,970 (38.0)	2,330 (33.5)	6,956 (38.2)		2,191 (42.2)	3,386 (39.9)	11,872 (39.1)	
High school or above	2,124 (40.0)	3,484 (50.3)	6,276 (39.4)		692 (13.3)	1,084 (13.2)	4,409 (14.4)	
Occupational status								
Employed	2,626 (50.3)	2,916 (42.2)	10,983 (60.0)	<0.001	4,702 (88.2)	6,047 (69.4)	24,701 (80.2)	<0.001
Unemployed	2,610 (49.7)	4,018 (57.8)	6,990 (40.0)		630 (11.8)	2,472 (30.6)	6,134 (19.8)	
Smoking								
Never smokers	3,912 (74.7)	5,070 (73.1)	12,403 (69.0)	<0.001	3,707 (69.5)	6,176 (72.9)	20,846 (67.4)	<0.001
Current smokers	1,230 (23.5)	1,672 (24.1)	4,924 (27.1)		1,564 (29.3)	2,229 (25.8)	9,198 (30.1)	
Ex-smokers	94 (1.8)	192 (2.8)	646 (3.8)		61 (1.1)	114 (1.3)	791 (2.6)	
Drinking frequency								
Almost never	4,395 (84.0)	6,442 (92.8)	16,334 (91.6)	<0.001	4,312 (82.2)	7,740 (92.4)	28,242 (92.0)	<0.001
1–2 times per week	664 (12.6)	266 (3.9)	921 (5.0)		701 (12.5)	309 (3.0)	1,442 (4.6)	
3 times or more	177 (3.5)	226 (3.3)	718 (3.5)		319 (5.3)	470 (4.6)	1,151 (3.5)	

^a^Numbers were unweighted, and percentages were weighted.

^b^P for differences between years within each subgroup using a chi-squared test.

The prevalences of NCDs in respondents aged 15 years and older are shown in Table 2. In urban regions, 23.9% of respondents reported having one or more chronic conditions in 2013, which is higher than that in 2008 (17.0%) and 2003 (12.8%); in rural regions, this proportion was 22.1% in 2013, which is also higher than that in 2008 (15.1%) and 2003 (10.9%). Chronic conditions were more common in urban regions than in rural regions for all waves. Over the last 10 years, a consistent increasing trend in the prevalences of chronic conditions was observed in all subgroups, except for respondents aged 15−34 years in rural regions.

**Table 2:** Crude prevalences of NCDs in 2003, 2008 and 2013.

	Urban			Rural		
	2003 Pr^†^, % (95% CI)	2008 Pr^†^, % (95% CI)	2013 Pr^†^, % (95% CI)	2003 Pr^†^, % (95% CI)	2008 Pr^†^, % (95% CI)	2013 Pr^†^, % (95% CI)
Total	12.8 (11.9−13.7)	17.0 (16.1−17.9)	23.9 (23.2−24.6)	10.9 (10.1−11.8)	15.1 (14.4−16.0)	22.1 (21.7−22.6)
Gender						
Men	11.4 (10.2−12.7)	16.1 (14.9−17.4)	23.3 (22.3−24.3)	9.4 (8.3−10.6)	13.3 (12.2−14.4)	19.6 (19.0−20.2)
Women	14.1 (12.9−15.5)	17.8 (16.5−19.1)	24.4 (23.5−25.4)	12.5 (11.2−13.9)	17.0 (15.9−18.3)	24.6 (23.9−25.3)
Age						
15−34	2.1 (1.5−2.9)	2.2 (1.6−2.9)	2.6 (2.1−3.1)	3.3 (2.6−4.2)	2.9 (2.3−3.7)	3.0 (2.6−3.4)
35−54	9.5 (8.3−10.9)	13.6 (12.5 −14.9)	16.4 (15.5−17.4)	12.3 (11.0−13.8)	15.1 (13.9−16.4)	17.7 (17.0−18.3)
55−64	27.6 (24.2−31.2)	31.8 (28.9−34.8)	37.4 (35.5−39.3)	22.9 (19.5−26.8)	27.3 (24.8−30.0)	35.2 (34.0−36.4)
65 years or older	33.0 (29.8−36.4)	44.1 (40.9−47.2)	54.8 (53.0−56.7)	23.3 (19.5−27.7)	30.9 (28.0−34.0)	45.7 (44.3−47.2)
Economic status						
Lowest 20%	8.5 (7.0−10.4)	14.4 (12.7−16.4)	20.7 (19.3−22.2)	9.7 (8.0−11.6)	14.6 (12.9−16.4)	22.7 (21.7−23.8)
Lower 20%	8.7 (7.2−10.6)	15.8 (13.9−17.8)	22.4 (21.0−24.0)	8.8 (7.2−10.8)	16.2 (14.4−18.1)	22.6 (21.6−23.7)
Middle 20%	12.1 (10.2−14.2)	16.9 (15.0−19.0)	23.0 (21.5−24.5)	11.0 (9.2−13.0)	12.4 (10.8−14.1)	21.4 (20.4−22.5)
Higher 20%	17.3 (15.1−19.8)	17.9 (16.0−20.0)	24.5 (23.0−26.1)	12.0 (10.1−14.2)	15.5 (13.8−17.5)	20.6 (19.6−21.7)
Highest 20%	17.5 (15.3−19.9)	19.8 (17.8−22.0)	28.9 (27.2−30.6)	13.2 (11.2−15.4)	17.0 (15.1−19.0)	23.3 (22.2−24.4)
Education level						
Primary school or below	24.3 (21.9−26.8)	34.1 (31.4−37.0)	38.3 (36.7−39.9)	15.9 (14.4−17.4)	22.2 (20.9−23.6)	30.8 (30.0−31.5)
Junior high school	10.2 (9.0−11.7)	16.0 (14.5−17.5)	20.4 (19.4−21.5)	6.9 (5.9−8.1)	9.2 (8.2−10.3)	15.8 (15.1−16.4)
High school or above	9.0 (7.8−10.3)	12.1 (11.1−13.2)	18.9 (17.9−20.0)	7.2 (5.4−9.5)	8.1 (6.5−10.0)	11.5 (10.6−12.5)
Occupational status						
Employed	7.4 (6.5−8.5)	10.7 (9.6−11.9)	15.1 (14.3−15.8)	11.4 (10.5−12.4)	15.1 (14.2−16.1)	20.3 (19.8−20.8)
Unemployed	18.3 (16.8−19.8)	21.5 (20.3−22.8)	37.1 (35.8−38.3)	7.2 (5.5−9.5)	15.2 (13.7−16.7)	29.7 (28.5−30.8)

^†^Prevalences were weighted.

**Fig 1:**
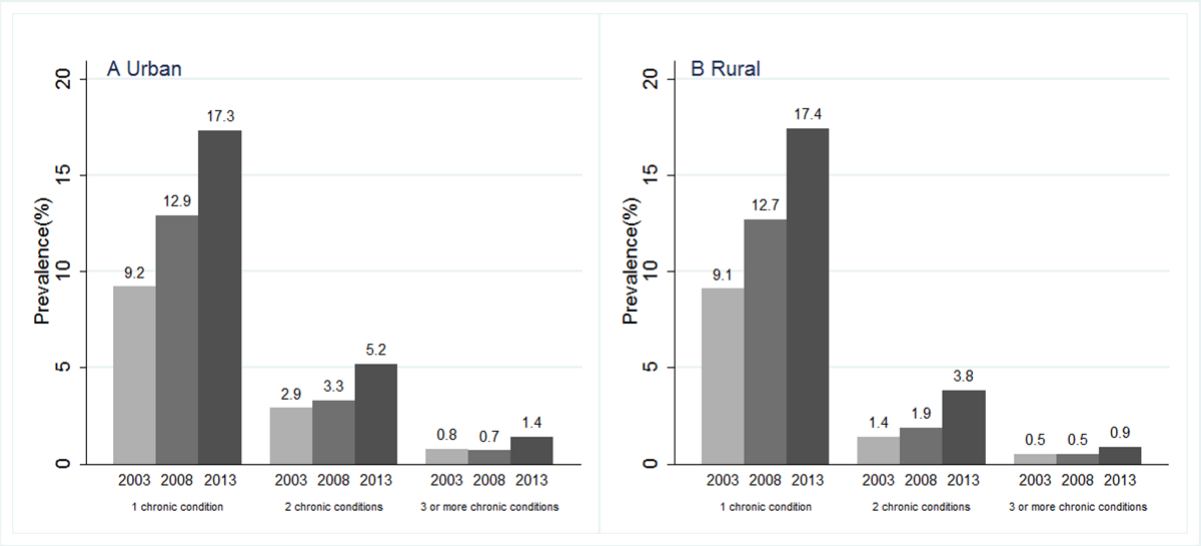
Crude prevalences of NCDs by number of chronic conditions in 2003, 2008 and 2013. Crude prevalences of NCDs by number of chronic conditions for each study year in urban regions are shown in panel A. Crude prevalences of NCDs by number of chronic conditions for each study year in rural regions are shown in panel B.

When studying the proportion of people with multiple chronic conditions (multimorbidity) across three waves, an increasing trend was observed (Fig 1). Specifically, we found that the prevalences of coexistence of several chronic conditions in urban regions almost doubled (increased from 3.7% to 6.6%), while the prevalences more than doubled (increased from 1.9% to 4.7%) in rural regions from 2003 to 2013.

Table 3 shows the prevalence rates of the top six chronic diseases and demonstrates the prevalences and trends of main chronic diseases between 2003 and 2013. Age-standardised prevalence of hypertension showed a significant increase over the decade (from 2.6% to 9.6% in urban regions and from 1.5% to 8.7% in rural regions, respectively) and is now the dominant chronic disease in both urban and rural regions, accounting for nearly 50% of total chronic diseases. A similar increasing trend was observed for diabetes (from 0.9% to 2.5% in urban regions and from 0.1% to 1.1% in rural regions, respectively). The percentage of chronic musculoskeletal disease in total showed a decrease, but prevalence rates increased from 1.8% to 2.5% in urban populations and from 2.0% to 3.0% in rural populations in the recent decade. Cardiovascular disease (CVD) is a class of diseases involving diseases of the heart, vascular diseases of the brain and diseases of blood vessels [24]. In urban regions, the prevalences and percentage of CVDs ranked second to hypertension. Compared with urban regions, rural residents had higher age-standardised prevalences of musculoskeletal diseases and chronic gastroenteritis/peptic ulcer, but they had lower age-standardised prevalences of hypertension, diabetes and cardiovascular diseases.

**Table 3:** Prevalences of the top six NCDs in 2003, 2008 and 2013^†^.

	2003			2008			2013		
Rank	NCDs	Pr^a^, %	%^b^	NCDs	Pr^a^, %	%^b^	NCDs	Pr^a^, %	%^b^
Urban									
1	CVD	3.0	21.2	Hypertension	5.8	32.4	Hypertension	9.6	43.7
2	Hypertension	2.6	18.1	CVD	3.2	18.6	CVD	2.9	13.8
3	MD	1.8	12.2	MD	2.2	11.4	Diabetes	2.5	11.7
4	CGPU	1.2	7.4	Diabetes	1.4	7.8	MD	2.5	10.2
5	Diabetes	0.9	5.9	CGPU	1.2	6.0	CGPU	1.0	3.7
6	COPD	0.8	5.9	CCGS	0.6	3.2	COPD	0.5	2.2
Rural									
1	CGPU	2.5	18.8	MD	2.7	17.4	Hypertension	8.7	42.9
2	MD	2.0	14.9	Hypertension	2.5	17.2	MD	3.0	13.8
3	Hypertension	1.5	10.9	CVD	2.0	13.7	CVD	2.2	10.7
4	CVD	1.5	10.8	CGPU	1.6	10.2	CGPU	1.5	6.4
5	Hepatitis/cirrhosis	0.7	5.6	COPD	0.9	6.2	Diabetes	1.1	5.4
6	COPD	0.7	4.6	Anaemia	0.6	3.9	COPD	0.6	3.0

^†^Other chronic diseases with lower prevalences were not listed in this table.

^a^Prevalences were adjusted according to the data of the 2010 national population census in Shaanxi Province.

^b^The percentage of chronic diseases in total for each year.

CVD, cardiovascular disease; MD, musculoskeletal disease; CCGS, cholecystitis/gallstone; CGPU, chronic gastroenteritis/peptic ulcer; COPD, chronic obstructive pulmonary disease.

To evaluate whether any of the age, gender and other socio-economic characteristics were associated with chronic conditions, multilevel and multivariate regression models were performed. The results are shown in Table 4. Higher age, higher economic status, low education level (primary school or below) and unemployed status in urban and rural areas, and female sex in rural areas were independently associated with chronic conditions.

**Table 4:** Association between chronic conditions and factors during 2003−2013.

	Urban PR (95% CI)^†^	Rural PR (95% CI)^†^
Survey year		
2003	1 (Ref)	1 (Ref)
2008	1.44 (1.11−1.85)**	1.29 (0.96−1.72)
2013	1.85 (1.46−2.34)**	1.61 (1.24−2.09)**
Gender		
Men	1 (Ref)	1 (Ref)
Women	1.03 (0.97−1.09)	1.23 (1.17−1.29)**
Age		
15−34	0.17 (0.14−0.21)**	0.22 (0.19−0.26)**
35−54	1 (Ref)	1 (Ref)
55−64	2.16 (1.99−2.35)**	1.87 (1.74−2.02)**
65 years or older	2.68 (2.43−2.95)**	2.08 (1.91−2.27)**
Economic status		
Lowest 20%	1 (Ref)	1 (Ref)
Lower 20%	1.05 (0.96−1.14)	1.14 (1.04−1.24)**
Middle 20%	1.09 (1.00−1.20)	1.15 (1.05−1.26)**
Higher 20%	1.19 (1.07−1.31)**	1.30 (1.18−1.43)**
Highest 20%	1.37 (1.24−1.52)**	1.53 (1.39−1.69)**
Education level		
Primary school or below	1 (Ref)	1 (Ref)
Junior high school	0.90 (0.85−0.97)**	0.81 (0.76−0.87)**
High school or above	0.86 (0.79−0.94)**	0.82 (0.73−0.92)**
Occupational status		
Employed	1 (Ref)	1 (Ref)
Unemployed	1.31 (1.22−1.42)**	1.18 (1.09−1.28)**

^†^Established by multilevel Poisson regression models with robust variance (three levels: individual, villages/communities and townships/sub-districts).

^Ref^Reference groups.

*P<0.05

**P<0.01.

In urban areas, in comparison with the 35- to 54-year-old group, the 55- to 64-year-old group (PR, 2.16; 95% CI, 1.99−2.35) and 65 years and older group (PR, 2.68; 95% CI, 2.43−2.95) showed a stepwise increase in the risk of NCDs. People with higher economic status (PR, 1.19; 95% CI, 1.07−1.31) and the highest economic status (PR, 1.37; 95% CI, 1.24−1.52) had a higher prevalence of NCDs than the poor. The prevalence of NCDs among the junior high school group (PR, 0.90; 95% CI, 0.85−0.97) and the high school or above group (PR, 0.86; 95% CI, 0.79−0.94) were significantly lower than the primary school or below group. The unemployed were 1.31 times (PR, 1.31; 95% CI, 1.22−1.42) as likely to have NCDs as the employed.

Similarly, in rural areas, the 55- to 64-year-old group (PR, 1.87; 95% CI, 1.74−2.02) and 65 years and older group (PR, 2.08; 95% CI, 1.91−2.27) had a higher prevalence of NCDs than the 35- to 54-year-old group. In comparison with the lowest economic group, the other four economic groups showed a stepwise increase in the risk of NCDs. The prevalence of NCDs among the junior high school group (PR, 0.81; 95% CI, 0.76−0.87) and the high school or above group (PR, 0.82; 95% CI, 0.73−0.92) were significantly lower than the primary school or below group. The unemployed were 1.18 times (PR, 1.18; 95% CI, 1.09−1.28) as likely to have the NCDs as the employed. The females were 1.61 times (PR, 1.61; 95% CI, 1.24−2.09) as likely to have the NCDs as the males.

Table 5 presents the differences between years of survey with the PRs estimated by multilevel and multivariate regression models, with 2003 denoting the reference point. We also attempted to explore how these changes differ by gender and age.

**Table 5:** Estimated relative change in NCDS in gender and age subgroups, 2003−2013.

		Gender Subgroup		Age Subgroup			
	Total PR (95% CI)^†^	Men PR (95% CI)^†^	Women PR (95% CI)^†^	Aged 15−34 PR (95% CI)^†^	Aged 35−54 PR (95% CI)^†^	Aged 55−64 PR (95% CI)^†^	Aged 65 years or older PR (95% CI)^†^
Urban: model 1^a^							
2003	1 (Ref)	1 (Ref)	1 (Ref)	1 (Ref)	1 (Ref)	1 (Ref)	1 (Ref)
2008	1.46** 1.12−1.92	1.47** 1.11−1.94	1.36* 1.03−1.79	1.13 0.54−2.39	1.55* 1.08−2.22	1.17 0.90−1.52	1.44** 1.10−1.87
2013	1.79** 1.39−2.30	1.82** 1.41−2.36	1.63** 1.26−2.09	1.30 0.65−2.62	1.85** 1.30−2.64	1.40** 1.12−1.74	1.78** 1.40−2.27
Urban: model 2^b^							
2003	1 (Ref)	1 (Ref)	1 (Ref)	1 (Ref)	1 (Ref)	1 (Ref)	1 (Ref)
2008	1.44** 1.11−1.85	1.45** 1.11−1.88	1.32* 1.01−1.72	1.26 0.60−2.67	1.54* 1.08−2.19	1.14 0.90−1.46	1.41** 1.10−1.81
2013	1.85** 1.46−2.34	1.87** 1.47−2.38	1.70** 1.35−2.15	1.30 0.65−2.62	1.95** 1.37−2.77	1.47** 1.20−1.80	1.82** 1.46−2.27
Rural: model 1^a^							
2003	1 (Ref)	1 (Ref)	1 (Ref)	1 (Ref)	1 (Ref)	1 (Ref)	1 (Ref)
2008	1.33 0.99−1.77	1.27 0.97−1.67	1.27 0.93−1.73	0.88 0.55−1.39	1.35 0.93−1.94	1.20 0.92−1.57	1.32 0.99−1.78
2013	1.63** 1.25−2.13	1.60** 1.26−2.02	1.59** 1.19−2.11	0.92 0.61−1.40	1.53* 1.10−2.14	1.53** 1.22−1.93	1.95** 1.53−2.49
Rural: model 2^b^							
2003	1 (Ref)	1 (Ref)	1 (Ref)	1 (Ref)	1 (Ref)	1 (Ref)	1 (Ref)
2008	1.29 0.96−1.72	1.22 0.93−1.61	1.25 0.92−1.71	0.96 0.63−1.46	1.30 0.90−1.87	1.15 0.88−1.49	1.26 0.95−1.69
2013	1.61** 1.24−2.09	1.56** 1.24−1.97	1.60** 1.21−2.11	1.08 0.72−1.63	1.52* 1.09−2.11	1.49** 1.19−1.86	1.86** 1.47−2.35

^†^Established by multilevel Poisson regression models with robust variance (three levels: individual, villages/communities and townships/sub-districts).

^a^Adjusted for gender and age.

^b^Additionally adjusted for economic status, education level and occupational status, with 2003 data used as a reference point in each subgroup.

^Ref^Reference groups

*P <0.05

**P <0.01.

The first model used chronic condition data adjusted for gender and age, and the second model additionally adjusted for socio-economic status. After adjusting for all variables used in the present analysis, the residents in 2013 were more likely to have chronic diseases than in 2003 (urban: PR, 1.85; 95% CI, 1.46−2.34; rural: PR, 1.61; 95% CI, 1.24−2.09).

There were subpopulation disparities in the trend of chronic diseases between 2003 and 2013. In urban regions, residents aged 35 to 54 years (PR, 1.95; 95% CI, 1.37−2.77) had higher adjusted PR for 2013/2003 than other corresponding groups; in rural regions, residents aged 65 years and above (PR, 1.86; 95% CI, 1.47−2.35) had higher adjusted PR for 2013/2003 than other age groups. The difference in the NCD prevalence between 2003 and 2013 was insignificant in the 15- to 34-year-old group in both urban and rural areas. Comparing the models, adjusting for social variables showed a slight decrease in the values of PRs for rural males, rural residents aged 35−54, 55−64 and 65 years or older.

## Discussion

The present study analysed the prevalences and trends of chronic diseases in Shaanxi Province, China, over one decade based on three representative cross-sectional surveys. We found that chronic diseases are common and have increased over time and that the prevalence of multimorbidity is growing. Additionally, the increase occurred largely in hypertension and diabetes during the last 10 years. The prevalence of hypertension in 2013 more than tripled since 2003, and that of diabetes increased nearly three times in urban areas and approximately ten times in rural areas. The results indicate that increased age, higher economic status, poor education, unemployment and female sex in rural areas were factors associated with the presence of chronic diseases. However, even after controlling for these established factors, the prevalences of chronic diseases were still higher in 2013 than in 2003. Age-related disparities in the trends of chronic diseases were evident in PR for 2013 vs 2003.

There are several possible explanations for the trajectory of increasing prevalences of chronic diseases. First, it can be suggested that the growing problem of population ageing may exacerbate the prevalences of chronic diseases, based on the results of higher NCD prevalence among older people, which is also consistent with the findings in previous studies. China’s aged population is growing rapidly while the number of young people is shrinking, due to the one-child policy implemented in 1979 and the increased life expectancy [25]. A huge demographic shift has been building up for decades. According to census figures, the proportion of the Chinese population aged over 65 years has grown to 8.87% in 2010 from 6.69% in 2000 [26]. In Shaanxi Province, this proportion has increased from 5.90% to 8.53% in that same period [27]. By 2050, the United Nations Population Division has projected that approximately 31% of the Chinese will be aged 60 years [28]. Additionally, life expectancy at birth in China has grown from nearly 71 years to 76 years between 2000 and 2011, although the economic level of China still lags behind developed countries [29]. The rising number of aged people has increased the incidence of NCDs associated with age, which may even worsen in the future [7].

The increase in age-standardised prevalences of NCDs indicates that a greater driving force has prompted the increase of NCDs. This driving force might be the increase in risk factors for chronic diseases. In China, prevalent smoking, drinking and other unhealthy behaviours deserve some attention. As the largest consumer and grower of tobacco in the world, China has 25% of the world’s smokers, with more than 288 million adult male smokers (52.9%) and 12.6 million adult female smokers (2.4%), and there are also 740 million nonsmokers exposed to passive smoking [30, 31]. In recent years, the Chinese government has made tremendous efforts in tobacco control [32], for example, increases in tobacco price and tax, bans on tobacco advertising and scenes of smoking in film and television, establishment of smoke-free environments in public places, and community outreach providing education about the adverse effects of smoking. Our results show that the proportion of current smokers steadily increased in urban regions and remained constant in rural regions over the last 10 years. However, the increasing proportion of ex-smokers indicates that tobacco control is improving slowly. More people have realised the hazards of tobacco and quit smoking. Similarly, our study shows that alcohol control has exhibited some signs of improvement in the last decade, especially low frequency of drinking. Previous studies have suggested that the main reason for changing unhealthy behaviours is because they are “bad for health” [33]; hence, known chronic conditions may prompt people to give up their unhealthy lifestyles and behaviours. In addition to smoking and drinking, excessive food intake is another important factor that should not be ignored, which was not included in our study. The prevalence of obesity has increased considerably [34, 35]. In 2012, 31.4% and 12.2% were overweight (BMI between 24 kg/m^2^ and 28 kg/m^2^) and obese (BMI ≥28 kg/m^2^) [36]; in 2002, these prevalences were 22.8% and 7.1%, respectively [37].

Most of the increase in chronic diseases between 2003 and 2013 was due to diabetes and, to a larger degree, hypertension, the latter accounting for nearly 50% of total chronic diseases. Hypertension-related risk factors coexist in many other chronic diseases, and hypertension-related cardiovascular morbidity is a crucial public health issue [38, 39]. Therefore, the results of the present study call for serious control action for hypertension. Additionally, chronic musculoskeletal diseases have increased in the recent decade. The WHO considers chronic musculoskeletal diseases to be a leading cause of morbidity and disability, incurring enormous healthcare expenditures [40].

Compared with previous studies, the disease prevalences were likely underestimated in our study. The self-report format implies that some people with undiagnosed chronic conditions were excluded. For example, a study using sphygmomanometer-based diagnosis indicated that 14% of Chinese adults suffered from hypertension in Western China in 2007 [11]. Another study, which used an oral glucose-tolerance test to diagnose diabetes, indicated that the prevalence of diabetes in China was 9.7% among individuals aged 20 years and older, which included self-report diabetes and undiagnosed diabetes using an oral glucose-tolerance test [10]. The self-report format of physician-diagnosed chronic diseases is lower in cost compared to anthropometric surveys, but it affects the accuracy of the analysis, particularly in previous years and in impoverished areas, where access to health services was problematic [41]. Therefore, in addition to the epidemiological increase in chronic diseases, our results are, in part, due to increased detection and diagnosis. The poorer populations have benefited in recent years from new healthcare reforms, such as the New Rural Cooperative (2003), the Urban Residents Health Insurance (2007), the Medical Assistance System (2005), Rural Cervical and Breast Cancer Screening Project (2009) and other basic public health policies. Early detection, diagnosis and treatment are important in order to improve the quality of life in patients with chronic diseases and decrease complications. Chronic disease surveillance systems and screening programmes should be established and improved.

The trend analyses between survey periods indicate that residents in 2013 were more likely to report chronic diseases than in 2003, after adjusting for gender, age and socio-economic status. Compared with the corresponding subgroups in 2003, the increase in chronic diseases occurred mainly in the middle-aged (35−54 years) and elderly (≥65 years) groups. The incidence of chronic diseases showed a trend in younger persons, especially among the middle-aged in urban areas. Recent studies warned that diabetes is developing in younger Asian people [42]. A previous research that studied mortality rates for selected chronic diseases in Xuzhou, China, suggested that more middle-aged people die from chronic diseases [43]. It might be related to unhealthy living and eating habits, considerable pressure, environmental deterioration and other factors. Hence, health concerns for the middle-aged are as important as for the elderly. Moreover, the results indicated that higher economic status, poor education and unemployment were factors associated with higher prevalences of chronic diseases, which were consistent with previous observations from low- and middle-income countries [44–48]. In China, which is now in a period of overall accelerative transformation, higher socio-economic groups appeared to be at a higher risk for chronic diseases, such as hypertension, diabetes and CVD, partly due to a Westernized lifestyle and diet [48]. The level of education, which is a key factor affecting health, has been highlighted by many health researchers. A lower educational level has been associated with lower health literacy and higher levels of disease risk factor [10]. Previous studies had found that unemployment was strongly associated with unhealthy behaviour, such as smoking and heavy drinking [49, 50].

Our study has several limitations. First, the study gathered data from three cross-sectional surveys, whereas a different sample volume existed between the surveys that may affect the ability of statistical inference to accurately estimate the prevalences. Additionally, the demographic structure of urban and rural regions is altering because of population migration and household registration policy in the 10-year span. It is possible that unintentional errors occurred when comparing the trend in chronic diseases, so a longitudinal design in future research may address the issue. Additionally, recall biases were unavoidable in questionnaire surveys, especially those questions involving the last year or 6 months before the survey. Second, some lifestyle characteristics (e.g., smoking and alcohol consumption) were excluded from the regression analysis owing to the endogeneity issue. Unhealthy lifestyles may lead to the development of NCDs, while being diagnosed with NCDs can also lead to changing to more healthy lifestyles. We include the smoking and alcohol consumption characteristics in the robust analysis, and our results remain stable. Third, the prevalences of NCDs were underestimated because undiagnosed diseases were not included due to the self-report format and because each respondent could enter a maximum of three chronic diseases. In practice, most NCDs are difficult to examine and diagnose among large populations using a standard approach and uniform diagnostic criteria. Several simple objective measures should be added to future investigations, such as measurements of blood pressure and blood sampling, and the symptom-based measures of diseases. Adding objective measures of health, we can not only obtain more accurate data on some special chronic diseases but we can also estimate the gap between self-reported diseases and criterion-based/symptom-based measures. Establishing a national chronic disease surveillance system and screening programme can compensate for the drawback of cross-sectional studies and questionnaires. It is worth mentioning that cancer is another growing health crisis over the past few in recent years [51], but it is hard to assess through community surveys. Most studies of cancer prevalence depend on the cancer registry data. In our data, the prevalence of cancer was approximately 0.2% in urban and rural areas in 2013. The main goal of our study was to explore the relative changes in the prevalences of chronic diseases during the last 10 years. For this study, we used the largest population surveys of NCDs implemented in this region, with random multistage stratified cluster sampling method used for sample representativeness and sampling weights used in the analysis for reflecting the survey methodology. Therefore, a certain amount of bias that existed within three surveys could, to some extent, be offset. Overall, developing countries are undergoing health transitions, and such surveys, despite their limitations, can build an evidence base to understand the dynamics of NCDs [52, 53].

A national chronic disease surveillance system and screening programme should be established and improved to understand the distribution and tendency of chronic diseases and offer evidence for scientifically formulating and evaluating the effects of prevention and control actions.

## Conclusions

In summary, our study provides evidence that chronic diseases are highly prevalent and continuously increasing in the adult population in Shaanxi Province, China. Hypertension and diabetes have become major chronic diseases emphasising the need for policies aimed at prevention, detection and treatment. Chronic disease-related strategies should specifically address those with the highest increase in the prevalence of chronic diseases, such as the middle aged and elderly. Given its large population, China may bear a higher NCDs-related burden than any other country and face more difficulties and challenges. A national chronic disease surveillance system could contribute to a better understanding of the distribution and tendency of chronic diseases.

## Abbreviations

NCD: Non-communicable disease
NHSS: National Health Services Survey
NHPC: National Health Commission of the People’s Republic of China
PR: Prevalence ratio
CVD: Cardiovascular disease
MD: Musculoskeletal disease
CCGS: Cholecystitis/gallstone
CGPU: Chronic gastroenteritis/peptic ulcer
COPD: Chronic obstructive pulmonary disease
